# The Future of Vineyard Irrigation: AI-Driven Insights from IoT Data

**DOI:** 10.3390/s25123658

**Published:** 2025-06-11

**Authors:** Simona Stojanova, Mojca Volk, Gregor Balkovec, Andrej Kos, Emilija Stojmenova Duh

**Affiliations:** Faculty of Electrical Engineering, University of Ljubljana, Tržaška cesta 25, 1000 Ljubljana, Slovenia; simona.stojanova@fe.uni-lj.si (S.S.); mojca.volk@fe.uni-lj.si (M.V.); gregor.balkovec@fe.uni-lj.si (G.B.); andrej.kos@fe.uni-lj.si (A.K.)

**Keywords:** sustainable agriculture, irrigation prediction, internet of things, sensors, linear regression, LSTM

## Abstract

Accurate irrigation volume prediction is crucial for sustainable agriculture. This study enhances precision irrigation by integrating diverse datasets, including historical irrigation records, soil moisture, and climatic factors, collected from a small-scale commercial estate vineyard in southwestern Idaho, the United States of America (USA), over a period of three years (2017–2019). Focusing on long-term irrigation forecasting, addressing a critical gap in sustainable water management, we use machine learning (ML) methods to predict future irrigation needs, with improved accuracy. We designed, developed, and tested a Long Short-Term Memory (LSTM) model, which achieved a Mean Squared Error (MSE) of 0.37, and evaluated its performance against a simpler baseline linear regression (LinReg) model, which yielded a higher MSE of 1.29. We validate the results of the LSTM model using a cross-validation technique, wherein a mean MSE of 0.18 was achieved. The low value of the statistical analysis (*p*-value = 0.0009) of a paired *t*-test confirmed that the improvement is significant. This research shows the potential of Artificial Intelligence (AI) to optimize irrigation planning and advance sustainable precision agriculture (PA), by providing a practical tool for long-term forecasting and that supports data-driven decisions.

## 1. Introduction

Climate change has long been a subject of scientific observation, but its effects have become increasingly urgent in recent decades. The average temperatures since 2010 have been constantly increasing and the newest report from the World Meteorological Organization (WMO) confirmed that 2024 was the warmest year ever recorded, with the global average near-surface temperature being 1.55 °C above pre-industrial levels. Additionally, it marked the hottest decade on record [1].

The impact of climate change is extensive. Rising temperatures, extreme weather events, and shifting precipitation patterns are placing immense pressure on agriculture, a sector crucial for global food security [2]. Moreover, with the global population growing, the demand for food is increasing, leading to the adoption of intensive agricultural practices, involving the use of agrochemicals, extensive livestock farming, and the exploitation of water resources.

Among these challenges, climate-induced droughts and water scarcity pose the greatest threats [3]. They result in soil degradation and a reduction in arable land, as well as reduced crop production, leading to social and economic instability for farmers. This growing problem has been identified as a major global threat [4]. According to the Global Assessment of Land Degradation and Improvement (GLADA), a quarter of the world’s land area is now considered degraded [5]. Land degradation leads to mass migrations. A 2017 United Nations Environment Programme (UNEP) report [6] states that 500 million hectares of farmland have been abandoned due to drought and desertification, creating significant social and environmental challenges. These insights stress the urgent necessity for comprehensive and coordinated efficient water management actions to tackle climate change impacts, mitigate risks, and enhance the resilience of vulnerable communities worldwide [3].

In recent years, the internet of things (IoTs) has emerged as a prominent technology, connecting real-world objects to the internet. These devices enable remote monitoring and management of critical parameters, such as soil nutrients, water dynamics, pest control, and yield prediction, provide timely updates and enabling remote control. IoT systems represent a cornerstone of modern agriculture, optimizing energy usage, reducing costs, and paving the way for innovations like indoor vertical farming systems, by leveraging data from IoT stations [7]. Precision agriculture (PA) nowadays integrates IoT sensors, wireless networks, and Artificial Intelligence (AI) to support sustainable farming, by optimizing irrigation, fertilizer use, soil management, and pest control, and with that optimizes resource use and improves decision-making processes [8]. It requires the gathering of both real-time and historical data. As the use of precision agriculture practices has increased, the volume of unstructured data has grown substantially. Current research focuses on extracting valuable insights and knowledge from these datasets [9].

This research investigates the impact of integrating the IoT and AI-driven predictive models on improving irrigation management. Theoretically, this study advances research on precision agriculture and smart farming by demonstrating how deep learning (DL) techniques can be effectively applied in agro-environmental contexts. Practically, our approach facilitates more accurate and timely irrigation planning, thereby enhancing water use efficiency, supporting sustainable agricultural practices, and mitigating the effects of climate variability. The findings offer actionable insights for farmers and vineyard managers, agronomists, and technology developers, aiming to implement data-driven decision support systems in viticulture. Specifically, we present a Long Short-Term Memory (LSTM)-based machine learning (ML) model, designed to forecast future irrigation needs. Through this analysis, we provide several contributions on this topic: (i) we show that DL techniques are better than traditional ML models in regard to processing time-series sequential data, hence showing better overall performance results; (ii) the performance of the LSTM model was affected by the number of inputs, i.e., the dataset, meaning that integrating a more diverse dataset can affect the performance of the model; (iii) we address long-term irrigation prediction; and (iv) we focus specifically on the factor, irrigation prediction, instead of other influencing factors (such as soil moisture, which is the focus of many studies). By developing, training, and testing an LSTM model, we achieve a balance between the simplicity and performance of the model, achieving high predictive accuracy, even with a limited dataset. The reduced model complexity provides possibilities for easy implementation, making it suitable for real-world use. This work proves that effective irrigation forecasting can be achieved without the need for complex analyses, offering an efficient solution for farmers. This model will serve as a basis for further analyses in terms of calculating the environmental, economic, and social aspects of digitalization in agriculture.

By leveraging the IoTs and AI, we aim to contribute to sustainable agricultural practices by enhancing irrigation management. This paper represents an advancement of our previous research on digitalization in agriculture. Starting with a review of smart village policies and their role in fostering rural development [10], followed by an exploration of the challenges and opportunities for integrating the IoTs into the business models of small rural entities [11], we plan to conclude this research by assessing the social, economic, and environmental return on investment in agriculture, with the use of SEROI+ [12], a tool developed within the Interreg project, ERUDITE [13], incorporating the findings from this research.

The paper is organized as follows: in Section 2 we present the literature review, Section 3 represents the methodological part, explaining the model development and testing process, followed by the presentation of the results and discussion in Section 4. We finish by providing the conclusion of the study.

## 2. Literature Review

The field of the IoTs is well-studied and recent research addresses several key areas. Studies have explored enabling technologies, integration factors across various domains, and key application areas. Researchers have examined IoT architecture, new applications and challenges, noting a significant increase in sensor deployments over the past decade. Discussions have also covered the basic requirements, characteristics, and everyday uses of IoTs, along with protocols and enabling technologies. Detailed analyses have been conducted, including on the architecture, communication techniques, identifying techniques, sensing technologies, networking, and processing capabilities of the IoTs [14,15].

The concept now known as precision agriculture gained prominence in the United States of America (USA) during the 1980s and was widely adopted in the early 1990s. Before this terminology started to be widely used, terms such as “Site-Specific Crop Management” or “Site-Specific Agriculture” were commonly used to represent farming methods similar to those still in use today [16].

The rise in the publication of papers related to precision agriculture shows a significant increase since 1996 [17]. The early development of precision agriculture focused on increasing crop yields through nutrient and crop variation, not on environmental concerns [18]. Today, the environmental impact is a key focus of precision agriculture. The USA leads in regard to the adoption of precision agriculture technologies and also ranks first in regard to its inclusion in research publications [19]. Australia has also emerged as a significant player, adopting innovations such as sensors, AI, and robotics, transforming its agricultural landscape, by enhancing its efficiency and sustainability [20]. In Europe, higher adoption rates in terms of precision agriculture are primarily seen in developed countries like the United Kingdom (UK), France, and Germany [21,22,23].

Research explores various advanced technologies for optimizing irrigation, with several approaches emerging in recent studies. Some focus on IoT-enabled precision irrigation systems that integrate real-time sensor data with ML to optimize water distribution [24]. Others leverage 6G IoT networks for AI-driven autonomous irrigation systems, enabling faster and more reliable communication [25]. Others explore digital twin models, integrated with IoT sensors, which enable process simulation and predictive modeling to anticipate water demand [26]. Additional research highlights the value of real-time canopy temperature monitoring for water balance estimation [27], humidity forecasting to enhance irrigation planning [28], and the integration of fog computing with Deep Neural Networks (DNNs) to develop smart irrigation systems [29]. Very often, soil moisture prediction is used as a basis for forecasting irrigation requirements [30].

Irrigation prediction methods are mainly categorized into traditional statistical techniques [31,32,33,34] and ML approaches [35,36]. Traditional models often face challenges in regard to accurately predicting irrigation needs, due to the complex and nonlinear nature of influencing factors, such as weather conditions and crop water requirements, positioning deep learning as a promising solution for enhanced prediction accuracy [36].

Examining studies that integrate ML or precision irrigation management, we can see that all three primary types of ML paradigms, including supervised [37,38,39], unsupervised [40,41] and reinforcement learning (RL) [42], as well as deep learning [43], are used for predicting irrigation needs, by analyzing environmental and crop data. Algorithms such as linear regression (LinReg) [38], random forest (RF), Support Vector Machines (SVMs), Decision Trees (DTs), LSTM, and RL, are all widely used [44].

In [35], the authors integrate IoT sensor data on soil moisture, temperature, and air humidity with multiple ML models, including K-Nearest Neighbors (KNNs), Logistic Regression (LogReg), neural networks (NNs), SVMs, and Naïve Bayes (NB). Their results indicate that KNNs achieves the highest prediction accuracy. However, the study does not incorporate past irrigation data, which limits its ability to capture temporal dependencies.

In [36], a Bidirectional Long Short-Term Memory (BiLSTM)–Convolutional Neural Network (CNN)–Attention Model is introduced for irrigation volume prediction. This model combines BiLSTM and a CNN to capture both past and future context leveraging meteorological data, soil properties, and crop parameters. The integration of BiLSTM, a CNN, and an attention mechanism enhances prediction accuracy by effectively handling time-series data, while capturing local features. Among the models predicting long-term dependencies in a single direction (past to future), LSTM ranked as the most accurate, followed by the CNN, with the recurrent neural network (RNN) performing the worst.

A more practical approach is presented in [45], wherein the authors develop a framework called the Irrigation Factor (IF) to estimate crop irrigation needs. The IF is determined based on three key indicators: soil moisture, leaf area index, and evapotranspiration. An LSTM model is then used to predict future values for these indicators, establishing upper and lower thresholds for mean, minimum, and maximum irrigation needs. The final daily irrigation requirements are calculated by applying weighted factors to each indicator. However, similar to previous studies, irrigation data is not considered in this analysis.

In contrast, Ref. [46] presents an ML approach specifically designed for predicting irrigation timing in regard to greenhouse tomato cultivation. This study incorporates past irrigation data (past irrigation timing), along with environmental variables (temperature, humidity, and solar radiation) and plant water stress metrics, which reflect plant health. A random forest (RF)-based model is developed to replicate expert farmers’ decision making. To address the challenge of imbalanced irrigation datasets, the authors propose an undersampling technique that removes non-irrigation data near irrigation events, improving the model’s accuracy. Their method achieved a recall of 92%, making it one of the few studies that integrate past irrigation data into predictions. For future work, the authors suggest exploring deep learning models, such as LSTM networks, to better capture sequential and time-dependent irrigation patterns, an approach we adopt in our study to enhance the model’s predictive performance.

In [47], the authors establish a decision support system (DSS) for precision irrigation in vineyards, using an NN model to calculate a daily thermal crop water stress index (CWSI) for grapevine. More precisely, real-time sensor data were used, gathered and stored on a field-deployed data logger, which computed a daily thermal CWSI for grapevines, using an NN model, fed with real-time sensor inputs. The data logger, connected via a cellular modem, also hosted webpages, displaying the sensor’s data history. These webpages were accessible to vineyard managers through the use of cell phones or computers. The CWSI-based IoT DSS was implemented at two small-scale commercial estate vineyards in southwestern Idaho, USA, over four growing seasons. At each site, the vineyard irrigation manager used the DSS daily to plan the irrigation schedules. The data from this study will be utilized in this paper to predict the irrigation needs, based on the collected sensor data.

Lastly, the authors in [44] explore the integration of various ML models to optimize irrigation decision management. It reviews the trends, applicability, and deployment of ML techniques for sustainable irrigation practices, emphasizing their potential use by farmers. As next stages, the authors suggest future advancements by developing cost-effective, user-friendly tools to support small-scale farmers.

From the presented literature review, we can conclude that researchers from different parts of the world have worked on creating models for predicting crop irrigation volumes. While these studies showcase significant advancements, there are some limitations, leaving gaps in regard to addressing long-term irrigation needs. Also, the application of ML for irrigation prediction faces some challenges and needs further research and validation to be conducted in order to maximize its potential and enhance its maturity and scalability across various regions and sectors. Despite significant advancements, several challenges remain in regard to the application of the IoTs and ML for irrigation prediction, and they can be classified as follows:

1. IoT technology accessibility. IoT systems are often costly and inaccessible to small-scale farmers, posing a significant barrier to adoption. Additionally, many farmers may lack the technical expertise or resources needed to implement these systems effectively. Our previous study explored this challenge in greater depth and presents a case study on rural Digital Innovation Hubs (DIHs) as one of the possible solutions to providing farmers with the necessary support [11], bridging the gap between technology and end-user adoption.
2. Data limitations. Many studies face challenges related to data scarcity [48], including limited access to high-resolution and long-term datasets, which are essential for training reliable ML models. As a result, researchers use attention mechanisms to focus on the most important input factors, helping to improve prediction accuracy [36]. The lack of large-scale, high-quality datasets contributes to model overfitting and underfitting, ultimately affecting prediction accuracy [44]. Another critical limitation is that many models fail to account for errors or uncertainties in input data and predictions [49]. Imbalanced datasets further compromise model performance. Because of the lack of past irrigation data, many studies focus on soil moisture prediction, such as [50,51,52,53], to name but a few, rather than directly addressing irrigation volumes. Furthermore, most IoT-based irrigation systems rely on a single or a limited set of sensors, whereas integrating multiple data sources, such as IoT sensor readings and historical irrigation records, remains underexplored. The use of historical irrigation data for forecasting future irrigation needs remains uncommon, presenting an opportunity for further research and innovation.
3. A focus on short-term, real-time predictions. Many existing systems primarily focus on short-term, causal-based predictions, such as daily or weekly irrigation needs, without considering long-term irrigation scheduling over an entire growing season. Reactive irrigation strategies are often insufficient in mitigating climate risks. This is where AI becomes transformative, providing opportunities for exceling in regard to processing time-series data, making them appropriate for predicting future irrigation needs based on historical data. Addressing both immediate and future irrigation requirements remains a critical research gap in the development of smart irrigation systems, highlighting the need for more comprehensive, season-wide prediction models [48].
4. ML model-related challenges. Traditional neural networks, such as Artificial Neural Networks (ANNs) and CNNs, face significant challenges when predicting irrigation volumes. These models often struggle to capture long-term dependencies in crop growth data, have limited capabilities in regard to extracting local features from sequential data, and are highly sensitive to noise. Moreover, they lack an attention mechanism to prioritize important information, reducing prediction accuracy [36]. On the other hand, deep learning techniques have made notable advances in addressing some of these challenges; however, as the complexity of the task increases, these models still encounter issues, such as information loss and decreased prediction accuracy, indicating the need for further refinement to enhance their performance in regard to more complex scenarios [36].
5. Standardization and scalability. Significant gaps remain in regard to standardization, along with a clear need for adaptable solutions that can perform effectively across diverse environmental conditions and agricultural contexts. This highlights the necessity for further research to address the current barriers and develop more versatile and scalable approaches [54].

Through this study, we try to address some of the highlighted challenges. Here is a summary of the main contributions from this paper, as follows:

1. Comprehensive dataset. This research integrates different types of data, including past irrigation data, soil moisture, and the following climatic factors, namely air temperature, relative humidity, solar radiation, wind speed, and wind direction metrics. By capturing a wider range of variables, we strive to improve model versatility and accuracy.
2. A focus on irrigation prediction. Unlike previous studies that primarily focus on soil moisture prediction, our research directly addresses the more complex and practical problem of predicting irrigation volumes, offering a solution with greater applicability for farmers.
3. Long-term irrigation prediction. Our study emphasizes long-term irrigation scheduling over entire growing seasons, addressing a critical gap in the existing research. By capturing temporal dependencies in irrigation needs, our model provides a more comprehensive solution compared to short-term, real-time prediction systems.
4. Practical application. By focusing on long-term irrigation prediction, we would like to provide additional value from this research, namely the possibility for practical use. The end goal of this research is to use the prediction results to calculate the associated irrigation costs and, through this, assess the social, economic, and environmental implications of digitalization in agriculture. Adding insight from the practical implementation of the study bridges the gap between research and real-world applications, providing tangible value to farmers and policymakers. This model, thus, offers a direct, efficient, and practical approach to irrigation forecasting, maintaining the model’s simplicity and computational efficiency.
5. ML model improvements. Our study uses the LSTM approach to predict future irrigation needs. The application of LSTM is well-suited for predicting time-series data, particularly for irrigation forecasting, where temporal dependencies are crucial. As mentioned earlier, many studies focus on the use of this model for achieving better accuracy in the results. Furthermore, by choosing this model, we would like to offer and test a simpler approach, which is more computationally efficient for practical irrigation scheduling tasks. In order to ensure model robustness, we incorporate cross-validation and use a moving average to reduce noise in the irrigation data, enhancing the prediction reliability of raw sensor data.

In conclusion, this research offers a more comprehensive, practical solution for irrigation forecasting using LSTM, addressing both time-series dependencies and the complexities of agricultural data. The focus on predicting irrigation demand, as opposed to just moisture levels or real-time decisions, provides the opportunity for better water resource management in farming, affecting environmental and economic aspects. Addressing some of the current issues, we aim to provide an enhanced, more accurate, adaptive, and sustainable irrigation system, supporting global agricultural productivity and sustainability.

## 3. Materials and Methods

### 3.1. Sensor Technology and Data Collection

In order to conduct irrigation prediction using historical irrigation data, soil moisture, and climatic data, we aim to focus on the practical use of the approach for real-world irrigation management. The data used for this study were obtained from the paper entitled “A crop water stress index based internet of things decision support system for precision irrigation of wine grape” [47]. As explained in the literature review, this paper utilizes a CWSI-based IoT DSS to monitor and manage irrigation in two vineyards for a period spanning over four growing seasons. The data collected come from different IoT sensors installed in the vineyards. More precisely, a drip irrigation method was used for collecting irrigation data, with tubes hanging about 30 cm above the ground and drippers that were 76 cm apart. The released water quantity was 3.8 L per hour. Irrigation application was firstly measured with tipping-bucket rain gauges (RainWise, Boothwyn, PA, USA), recording data every 15 min and every 24 h, and then using flow meters (36MP.75RG.1, Netafim Irrigation, Inc., Fresno, CA, USA), recording data every 15 min. A soil moisture sensor (Drill and Drop, SenTek Sensor Technologies, Stepney, SA) was installed in the vine row. These parameter data were recorded every 30 min, and the measurements were taken at different levels, from 10 cm to 120 cm in depth. The collection of data on the climatic parameters included the air temperature, relative humidity (HMP50 temperature and humidity probe, Campbell Scientific, Logan, UT, USA), wind speed, and direction (034B, Met One Instruments, Grants Pass, OR, USA) at a height of 2.5 m, measured every minute and recorded as 15 min averages. The collected data were saved on a data logger (CR6, Campbell Scientific, Logan, UT, USA) placed in the vineyards. The data logger included a machine-to-machine cellular modem (either RavenXTA CDMA or RV50 Sierra Wireless AirLink, Campbell Scientific Inc., Logan, UT, USA). Data management software (LoggerNet 4.5, Campbell Scientific Inc., Logan, UT, USA) was used to retrieve the data and the vineyard managers could access the data through a website, designed and published using data logger webpage software (RTCM Pro Development 4.3.3.6, Campbell Scientific Inc., Logan, UT, USA). The data were accessible during the daily working hours. During the night, in order to save battery and for data security reasons, the cellular modem was turned off. The data logger battery was powered with a 20 watt solar panel [47].

For this analysis, the available data comes from one vineyard, collected for the period of 2017–2019. The features selected for the analysis were derived from the following variables:

1. The soil moisture recorded volumetric water content (SVWC) at depths ranging from 10 cm to 120 cm (SVWC 10 cm, SVWC 20 cm, …, SVWC 120 cm), representing the vertical soil moisture profile;
2. Climatic variables, namely air temperature (AirTC_Avg), relative humidity (RH_Avg), solar radiation (RS_Avg), wind speed (WS_ms_Avg), and wind direction metrics (WindDir_Avg, WindDir_StDev);
3. Irrigation records, namely the amount of water applied for maintaining crop health.

### 3.2. Data Preprocessing and Dataset Construction

The data were stored in multiple Excel files, with each year’s data recorded separately. To streamline the data processing, the raw data files were first split into individual sheets corresponding to different variables, followed by cleaning and formatting. Non-essential rows (headers, units) were removed, and missing or irregular timestamps were interpolated or discarded, ensuring the temporal integrity of the dataset.

**Figure 1:**
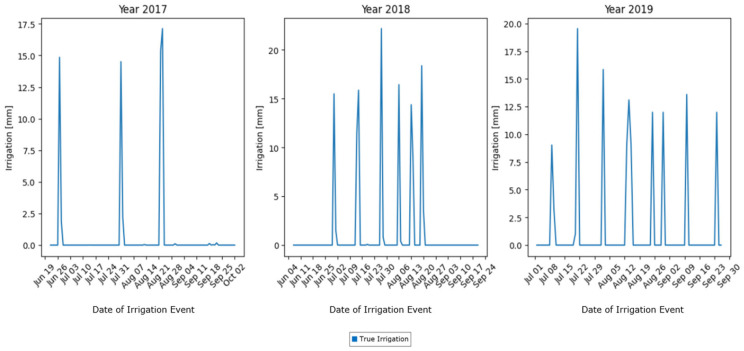
True irrigation data for years 2017, 2018, and 2019.

**Figure 2:**
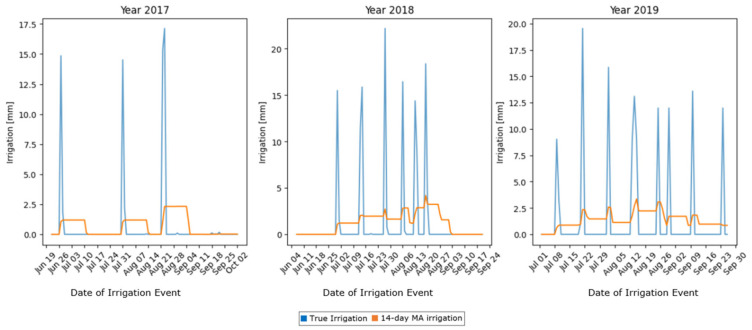
The 14-day MA of the irrigation data for years 2017, 2018, and 2019.

The irrigation data exhibited high variability, with infrequent, but significant, spikes in the data. For instance, irrigation data were recorded only during 3 to 8 days per season, resulting in a sparse dataset, wherein most values were zero (Figure 1). To address this sparsity and improve the predictability of the target variable, a 14-day moving average (MA) was applied. The choice of a 14-day MA window was driven by the nature and quality of the data. We tested different window sizes. A 7-day MA still produced so-called “jagged” data and, hence, was not adequate. On the other hand, longer windows, such as 20-day or 30-day Mas, provided smoother signals, but reduced the number of data points. This data reduction was seen as a limitation to the further training and testing of the model. Hence, the 14-day MA provided the best possible solution, transforming the target into a continuous and stable signal, without compromising efficient model development. The smoothed target variable enabled the model to better learn the irrigation patterns by reducing noise, while preserving the overall trends (Figure 2).

The data for different features were collected at different sub-daily intervals (e.g., 15 min or 30 min). To align these data with the daily target labels, the feature data were aggregated into daily averages. This aggregation reduced the dimensionality of the input space, while preserving the relevant information.

An important step was to match the input data (features) with the irrigation needs (target). Since the features were averaged daily and the irrigation data were smoothed, the timing had to be adjusted. To make sure the data for day t (today) predicted the irrigation need for day t + 1 (tomorrow), the target data (irrigation need) were shifted back by one day. This ensured that the input data and the target were properly aligned. The final target variable (label) was the smoothed irrigation requirement offset by one day to ensure the predictions were made using the features from the preceding day.

Missing values in the dataset, identified as null entries or gaps, were handled through interpolation or row removal. Rows with null target values or critical features were dropped to ensure model integrity. The processed datasets for 2017, 2018, and 2019 were merged into a single dataset of 291 daily entries for further analysis.

### 3.3. Model Development

#### 3.3.1. Baseline Model: Linear Regression

**Figure 3:**
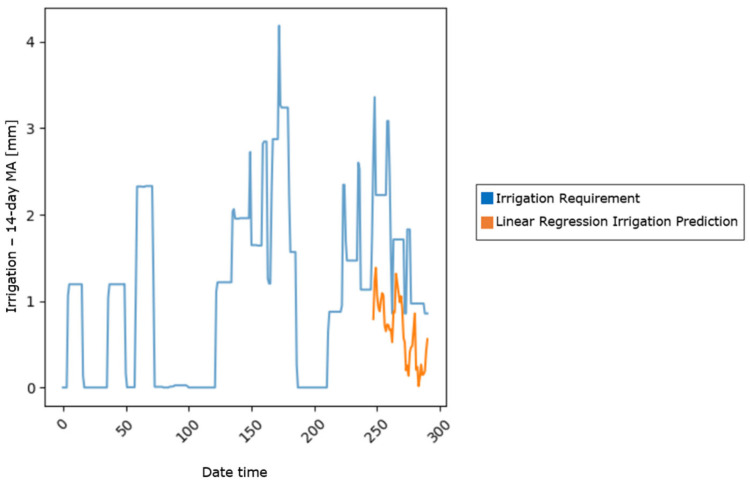
Linear regression model prediction.

As an initial benchmark, a multiple linear regression model was developed using three core features: soil moisture at a 10 cm depth (SVWC 10 cm), air temperature (AirTC_Avg), and relative humidity (RH_Avg). These features were selected based on their direct influence on the soil water content and evapotranspiration. For the data preprocessing in regard to the linear regression model, the features were standardized using StandardScaler to normalize their ranges and ensure comparability. The dataset was then split chronologically, with 85% allocated for training and the remaining 15% reserved for testing, maintaining the temporal sequence of the data. The baseline model achieved a test set MSE of 1.29, establishing the benchmark for model improvement. The model is presented in Figure 3.

#### 3.3.2. Three-Feature LSTM Model

This model used the same features as the baseline regression model, namely the soil moisture at a 10 cm depth (SVWC 10 cm), air temperature (AirTC_Avg), and relative humidity (RH_Avg). The features were reshaped to include a time dimension, allowing the model to process one day of features at a time. Due to the limited dataset and in order to reduce the risk of overfitting, the LSTM model architecture consisted of a single LSTM layer with 4 units, followed by a dense output layer for predictions. Both the input features and labels were normalized using StandardScaler to ensure consistency. The model was trained for 30 epochs, with a batch size of 1, using the Adam optimizer to achieve efficient learning. The data were split chronologically into 85% training and 15% testing sets to preserve the temporal order. The input features and labels were scaled separately to enhance model performance during training.

**Figure 4:**
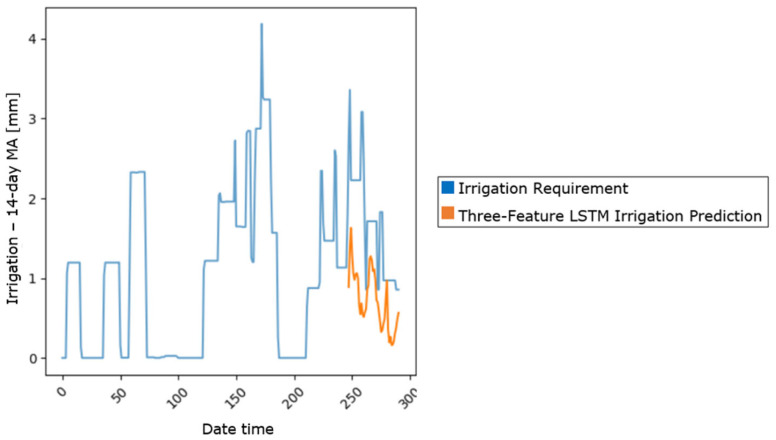
Three-feature LSTM model prediction.

The three-feature LSTM model demonstrated a clear reduction in the training loss over the 30 epochs, indicating effective learning. It achieved a test MSE of 1.21, slightly outperforming the baseline linear regression model, which had an MSE of 1.29. However, the higher test loss compared to the training loss suggested that the model experienced overfitting, likely due to its simplicity (Figure 4).

#### 3.3.3. Full-Feature LSTM

This advanced model extended the basic architecture by incorporating all 19 features and a 7-day lookback window in order to enhance the model performance and, at the same time, provide sufficient historical data to capture key temporal patterns, while maintaining relevance in regard to real-world irrigation practices. Each input into the LSTM consisted of a sequence of daily feature values over the past 7 days, providing temporal context for the irrigation predictions. The full-feature LSTM achieved an MSE of 0.37 based on the test set, a significant improvement over the baseline model and the three-feature LSTM model. Same as above, the model was also trained for 30 epochs, with a batch size of 1, using the Adam optimizer to achieve efficient learning.

**Figure 5:**
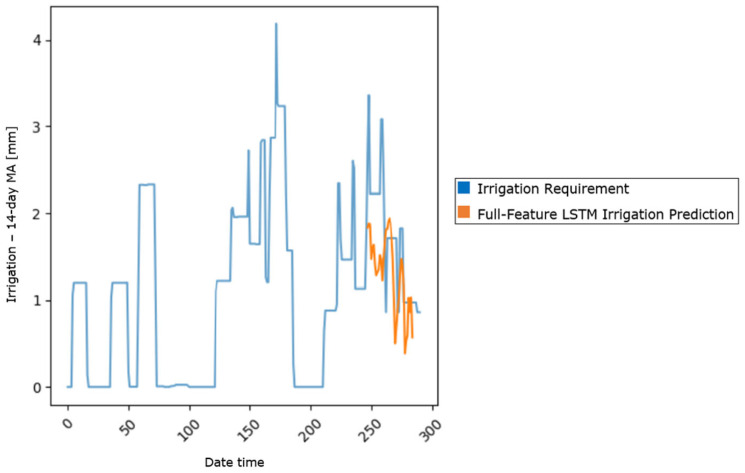
Full-feature LSTM model prediction.

The full-feature LSTM model addressed the limitations of the three-feature LSTM version by utilizing a wider range of features and incorporating temporal patterns through the lookback mechanism. This approach led to a significant improvement in performance, with the test MSE dropping to 0.37, outperforming both the basic three-feature LSTM and the linear regression model, an indication of improved accuracy (Figure 5). Despite these improvements, some degree of overfitting persisted, as evidenced by the discrepancy between the training and test loss values. An overview of the achieved performance metrics for each of the three models is presented in Table 1.

In conclusion, the basic three-feature LSTM model provided a foundational framework, but was limited by its simplicity and lower accuracy. The full-feature LSTM model, with its richer feature set and temporal context, demonstrated a superior predictive capability and highlighted the importance of incorporating historical patterns in time-series analysis. However, addressing overfitting through the use of techniques like regularization or by increasing the dataset size could further enhance the robustness of these models.

### 3.4. Cross-Validation Approach

To robustly evaluate the models, a 5-fold cross-validation strategy was applied to the baseline linear regression model and the full-feature LSTM model. Cross-validation involves splitting the dataset into multiple subsets, or “folds,” iteratively using one fold as the test set, while training the model based on the remaining folds. This ensures that every data point is used for both training and evaluation, providing a more reliable assessment of model performance compared to a single train–test split.

For the baseline linear regression model, the dataset was randomly split into five folds. The features were normalized for each fold, and the models were trained and evaluated iteratively. For the full-feature LSTM model, sequential folds were used to preserve the temporal order, critical for time-series data. The training involved creating sequences of features for a 7-day lookback window and scaling the inputs and outputs.

The results showed that the baseline model achieved a mean MSE of 0.83, with a standard deviation of 0.16. In comparison, the LSTM model demonstrated significantly improved performance, achieving a mean MSE of 0.18, with a standard deviation of 0.06 (Table 2).

The cross-validation results demonstrate that the full-feature LSTM model has a much smaller range, indicating more consistent performance across different data splits, highlighting the model’s robustness, validating the potential of LSTM networks for practical applications in precision agriculture.

### 3.5. Statistical Significance

A paired *t*-test was conducted to compare the cross-validation results of the baseline and LSTM models. The *t*-test is particularly suitable for dependent samples, such as those derived from cross-validation, as it accounts for the paired nature of the observations. The analysis yielded a t-statistic of −8.79 and a *p*-value of 0.0009. The *p*-value, being significantly less than the threshold of 0.05, indicates that the performance improvement of the LSTM model over the baseline model is statistically significant. This confirms that the observed gains are unlikely to be due to random chance.

**Figure 6:**
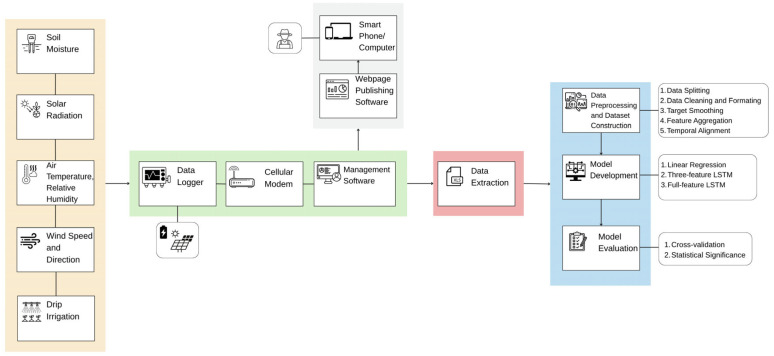
Network architecture diagram.

The whole process, explained in this section, from sensor data collection to ML model development and evaluation, is presented in the network architecture diagram shown in Figure 6.

## 4. Results and Discussion

LSTM is a type of deep learning model. It belongs to the family of recurrent neural networks and, in particular, it is designed to handle sequential data and long-range dependencies [55]. It demonstrates strong predictive capabilities and is extensively applied in regard to various hydrologic time-series predictions [56].

Irrigation volume prediction is a complex task that involves the forecasting of the water requirements of crops based on different datasets, such as weather patterns, soil conditions, and historical data. Traditional neural networks face several limitations in regard to capturing local fluctuations in data, adapting to the importance of different sequence parts, and maintaining long-term dependencies [57]. In regard to irrigation prediction, data fluctuations, such as sudden weather changes, play a significant role in accurate forecasting. In the context of irrigation prediction, certain periods in the sequence, such as droughts or sudden weather events, are more important for making accurate predictions. Traditional models often fail to capture these local features due to their limited perception of sequential data. LSTM models, however, process sequences step by step, enabling them to capture both local and long-term dependencies [58], offering significant advantages in regard to overcoming these challenges, making them a promising solution for irrigation prediction [59].

LSTM models are specifically designed to address the vanishing gradient problem and effectively maintain long-term dependencies through their unique architecture [60]. The cell state in an LSTM model allows the network to retain crucial information across different time steps, while the gates regulate information flow, allowing the model to remember and update past inputs as needed and to focus on the most relevant data. This capability is particularly beneficial in irrigation prediction, where long-term trends (e.g., seasonal changes) are critical [57]. The enhanced local perception capability enables LSTM-based models to effectively learn from detailed temporal patterns, ensuring that the model can concentrate on significant time periods, improving its ability to make accurate predictions [60,61].

Our LSTM model, incorporating a 7-day lookback window and full feature set, achieved a test MSE of 0.37, significantly outperforming the baseline linear regression model (MSE: 1.29). Cross-validation further confirmed the robustness of the LSTM model, with a mean MSE of 0.18 and a standard deviation of 0.06. A paired *t*-test comparing the baseline and LSTM model yielded a t-statistic of −8.79 and a *p*-value of 0.0009, confirming the LSTM model’s performance improvement as statistically significant. The LSTM model’s superior accuracy is attributed to its ability to capture temporal dependencies inherent in irrigation data. Unlike linear regression, which treats daily inputs as independent, the LSTM model’s gated architecture (input, forget, and output gates) explicitly models how irrigation needs depend on historical sequences of soil moisture, climate conditions, and past irrigation events. The 7-day lookback window enabled the model to recognize patterns, such as cumulative drought effects or post-rainfall recovery, critical for long-term forecasting. This aligns with established applications of LSTM models in hydrology, wherein temporal coherence is paramount.

While the LSTM model is more complex than linear regression, it remains practical for real-world use. It makes predictions quickly (~0.2 s each) on standard computers. Although training takes longer (30 passes of the data), this process can be further improved using a Graphics Processing Unit (GPU) support. It represents a valuable future improvement of the model, bearing in mind the improved accuracy (71% fewer errors than linear regression) of the model. This improvement could make the model even faster, while maintaining its accuracy.

Using simpler ML models, such as LogReg [62], KNNs [35], and Support Vector Regression (SVR) [63], are more common in the literature on smart irrigation prediction. However, studies that use LSTM models show that LSTM models outperform other models, as in [64] for example. In [36], the authors use an advanced BiLSTM–CNN–Attention Model approach, integrating multiple deep learning layers, which is useful for large agricultural irrigation forecasting. In our study, we focus on a specific, small-scale area, in a concrete vineyard, making further use of the collected data. By focusing on the LSTM model, we reduce the model complexity, making it more easy to implement and use in the real world, and despite the smaller dataset, the full-feature LSTM model demonstrates high predictive accuracy. These contributions show that good irrigation prediction can be achieved using simpler DL models, especially when they are applied to specific crops, wherein interpretability and easy implementation are important. Whereas a more practical approach is presented in [45], the authors develop an LSTM model for each of the three indicators determined for predicting irrigation and the results show a range of mean, minimum, and maximum levels of irrigation requirements, whereas we combine all the inputs into a single LSTM model. Additionally, in order to address the challenge of imbalanced irrigation data in our LSTM model, we use a 14-day MA smoothing technique, instead of an undersampling method, avoiding the loss of important information. This captured irrigation trends instead of separate events, improving the learnability of the signal. Compared to undersampling, our smoothing approach provided a richer and more informative target representation, supporting more effective model training and improved predictive performance. The alternative approach, using undersampling, was paired with a random forest model in [46]. Although undersampling is an appropriate technique for random forest models, and it provides more balanced data, without compromising the model’s assumptions or architecture, we use smoothing, which, in contrast, modifies the nature of the target variable and temporal dynamics, which random forest does not inherently exploit. Through this study, we confirm that compared to traditional ML models, DL models significantly increase the ability to process big data, resulting in better model performance [65].

While the analysis in this study demonstrates significant improvements in irrigation prediction using an LSTM model, there are several areas where improvements could be made to enhance its performance, accuracy, and applicability in real-world agricultural settings, as follows:

- Limited data variety and scope. The model was trained and tested on a dataset consisting of soil moisture, climatic variables, and irrigation data that were collected over three years. While the dataset provides useful information, it is limited to a single vineyard over three years, which may affect the model’s generalizability. Future research could expand the dataset to include multiple vineyards and longer time periods.
- Data feature selection. Although the model utilizes a comprehensive set of features (soil moisture, climate data, and irrigation data), the process could be further refined through the inclusion of additional factors, such as crop-specific characteristics like growth stage, water needs, and soil texture. The inclusion of additional environmental variables may enhance the prediction performance.
- Overfitting and model complexity. One common challenge when using deep learning models like LSTMs is the potential for overfitting, especially when the training data are limited or noisy. Regularization techniques, such as dropout, or more advanced methods, like attention mechanisms, could be explored to mitigate overfitting [66]. In order to avoid overtraining, rather than setting a fixed number of epochs, early stopping could be considered, as well as experimenting with learning rate schedules to identify optimal values.

In summary, while the model demonstrates the potential for accurate irrigation forecasting, addressing these limitations through expanded data, improved preprocessing, more robust regularization, and enhanced interpretability could significantly enhance its performance and applicability in precision agriculture. By addressing these areas, future iterations of the model could provide more accurate, efficient, and adaptable irrigation solutions, improving water management in agriculture and contributing to sustainable farming practices. Furthermore, the use of these inputs, soil moisture, climate data, and irrigation data, provides the possibility for broader applicability of the model in regard to different crop types and locations, if a similar sensor structure is available. The inclusion of additional environmental variables may not only enhance the prediction performance of the model, but by adjusting the features to suit specific crop types, the model also has the potential to generalize its predictions to other crops and regions. Additionally, the LSTM model can learn temporal patterns, with this learning about other crop-related or location-related specifications adding to the model’s generalizability.

## 5. Conclusions

With the ongoing trend in the use of IoT sensors for sustainable agriculture, the goal of this paper was to design, train, and test an LSTM model for predicting irrigation requirements, contributing to the theoretical and practical research on sustainable agriculture and precision irrigation. To do so, we used a dataset that consisted of historical irrigation, soil moisture, and climatic factors. The methodology incorporated advanced preprocessing techniques, such as smoothing the irrigation data with a moving average and the temporal alignment of features and labels, to handle the challenges posed by sparsity and variability in the dataset. After preparing the final dataset, we first developed a baseline linear regression model, to serve as a starting point and a basis for the comparison. Then, we built a simpler, three-feature LSTM model (incorporating three inputs from the dataset), and, at the end, we developed a more complex full-feature LSTM model (incorporating all the inputs from the dataset). The linear regression model provided a foundation for the comparison, achieving an MSE of 1.29, showing limited predictive accuracy. The LSTM models significantly outperformed the baseline model, with the full-feature LSTM model incorporating a 7-day lookback window, achieving an MSE of 0.37. Cross-validation further validated the robustness of the LSTM model, yielding a mean MSE of 0.18, with low variance (standard deviation: 0.06). The statistical analysis confirmed the improvement as highly significant (*p*-value = 0.0009), emphasizing the capability of LSTM models to capture temporal dependencies and complex interactions among features.

This study makes several key contributions to the field of precision irrigation. We focus on factor irrigation prediction by integrating diverse datasets, including historical irrigation records, soil moisture, and climatic factors. We address the data limitations highlighted in regard to previous research, by focusing on long-term irrigation prediction. Also, we demonstrate the feasibility and effectiveness of deep learning models, particularly LSTM networks, in predicting irrigation needs, by presenting a simpler practical solution.

By adjusting the features to suit specific crop types and local variables, the model has the potential to generalize its predictions to other crops and regions. This flexibility allows for its broader application in precision agriculture, helping to optimize irrigation across diverse agricultural settings. Additionally, transfer learning offers a promising opportunity to apply knowledge gained from one vineyard to another. By leveraging the data and insights from an established model, it is possible to transfer learned patterns and features to a new vineyard, reducing the need for extensive retraining.

Despite these promising results, the study identified challenges such as overfitting and limited data availability. Addressing these challenges through regularization techniques, additional data sources, and broader environmental variables could enhance the model’s generalization and applicability.

In conclusion, this research highlights the potential of advanced ML techniques in optimizing irrigation planning strategies, paving the way for sustainable agricultural solutions that balance productivity with resource conservation.

## Figures and Tables

**Table 1:** Performance metrics for linear regression, three-feature, and full-feature LSTM models.

Model	MSE	Std. Deviation
Linear Regression	1.29	0.33
Three-feature LSTM Model	1.21	0.25
Full-feature LSTM Model	0.37	0.59

**Table 2:** Cross-validation performance metrics foe linear regression model and full-feature LSTM model.

Model	Mean MSE	Std. Deviation
Linear Regression	0.83	0.16
Full-feature LSTM	0.18	0.06

## Data Availability

The data used in this study were obtained from [King, B.A., & Shellie, K.C. (2023) . A crop water stress index based internet of things decision support system for precision irrigation of wine grape. Smart Agricultural Technology, 4, 100202] [47] and are available upon request.

## Abbreviations

The following abbreviations are used in this manuscript:

ML	Machine learning
LSTM	Long Short-Term Memory
MSE	Mean Squared Error
LinReg	Linear Regression
AIPA	Artificial Intelligence Precision Agriculture
WMO	World Meteorological Organization
GLADA	Global Assessment of Land Degradation and Improvement
UNEP	United Nations Environment Programme
IoTs	Internet of things
DL	Deep learning
USA	United States of America
UK	United Kingdom
RF	Random forest
SVMs	Support Vector Machines
DTs	Decision Trees
RL	Reinforcement Learning
KNNs	K-Nearest Neighbors
LogReg	Logistic Regression
NNs	Neural networks
NB	Naïve Bayes
BiLSTM	Bidirectional Long Short-Term Memory
CNN	Convolutional Neural Network
RNN	Recurrent neural network
IF	Irrigation Factor
DSS	Decision support system
CWSI	Crop water stress index
DIHs	Digital Innovation Hubs
ANNs	Artificial Neural Networks
SVWC	Soil volumetric water content
MA	Moving average
GPU	Graphics Processing Unit
SVR	Support Vector Regression
